# The Effects of Airway Pressure Release Ventilation on Pulmonary Permeability in Severe Acute Respiratory Distress Syndrome Pig Models

**DOI:** 10.3389/fphys.2022.927507

**Published:** 2022-07-22

**Authors:** Jiangli Cheng, Jing Yang, Aijia Ma, Meiling Dong, Jie Yang, Peng Wang, Yang Xue, Yongfang Zhou, Yan Kang

**Affiliations:** Department of Critical Care Medicine, West China Hospital of Sichuan University, Chengdu, China

**Keywords:** acute respiratory distress syndrome, mechanical ventilation, airway pressure release ventilation, low tidal volume, pulmonary permeability

## Abstract

**Objective:** The aim of the study was to compare the effects of APRV and LTV ventilation on pulmonary permeability in severe ARDS.

**Methods:** Mini Bama adult pigs were randomized into the APRV group (*n* = 5) and LTV group (*n* = 5). A severe ARDS animal model was induced by the whole lung saline lavage. Pigs were ventilated and monitored continuously for 48 h.

**Results:** Compared with the LTV group, CStat was significantly better (*p* < 0.05), and the PaO_2_/FiO_2_ ratio showed a trend to be higher throughout the period of the experiment in the APRV group. The extravascular lung water index and pulmonary vascular permeability index showed a trend to be lower in the APRV group. APRV also significantly mitigates lung histopathologic injury determined by the lung histopathological injury score (*p* < 0.05) and gross pathological changes of lung tissues. The protein contents of occludin (*p* < 0.05), claudin-5 (*p* < 0.05), E-cadherin (*p* < 0.05), and VE-cadherin (*p* < 0.05) in the middle lobe of the right lung were higher in the APRV group than in the LTV group; among them, the contents of occludin (*p* < 0.05) and E-cadherin (*p* < 0.05) of the whole lung were higher in the APRV group. Transmission electron microscopy showed that alveolar–capillary barrier damage was more severe in the middle lobe of lungs in the LTV group.

**Conclusion:** In comparison with LTV, APRV could preserve the alveolar–capillary barrier architecture, mitigate lung histopathologic injury, increase the expression of cell junction protein, improve respiratory system compliance, and showed a trend to reduce extravascular lung water and improve oxygenation. These findings indicated that APRV might lead to more profound beneficial effects on the integrity of the alveolar–capillary barrier architecture and on the expression of biomarkers related to pulmonary permeability.

## Introduction

Acute respiratory distress syndrome (ARDS) is a serious clinical disease with high incidence and mortality. For critically ill patients with ARDS, mechanical ventilation (MV) is an essential life-saving intervention (Fan et al., 2018). However, MV can also cause or exacerbate lung injury, a syndrome known as ventilator-induced lung jury (VILI), which might aggravate the progression of ARDS and lead to adverse clinical outcomes (Slutsky and Ranieri, 2013; Bates and Smith, 2018).

ARDS is characterized by an increased permeability of the alveolar–capillary barrier which facilitates influx of protein-rich fluid, neutrophils, erythrocytes, and other macromolecules into alveolar space, leading to pulmonary edema and consolidation, thus resulting in hypoxemia due to the impairment of the gas exchange (Albert, 2012; Wang et al., 2017). Increased pulmonary permeability is an important pathogenesis in the development of ARDS, and MV-inducing lung injury probably resulted from the increased permeability of the alveolar–capillary barrier (Tsukimoto et al., 19851991; Thompson et al., 2017). Therefore, a mechanical ventilation strategy which could decrease pulmonary permeability may prevent the progression of ARDS and protect the lung from injury.

The low tidal volume (LTV) ventilation strategy used for ARDS has been widely accepted during the past 20 years, but the mortality still remains unacceptably high, especially for severe ARDS patients. Studies have found that LTV ventilation also could lead to VILI due to the heterogeneity injury of lungs in ARDS patients (Terragni et al., 2007; Bellani et al., 2016; Kollisch-Singule et al., 2016; Villar et al., 2016; Fan et al., 2017). Airway pressure release ventilation (APRV) used earlier in ARDS patients can gradually open the collapsed alveolar through extending the phase of high pressure and also can avoid the alveolus to collapse by shortening airway pressure release time. Additionally, it allows patients to breathe spontaneously, which may improve the patient–ventilator interaction and might compensate the negative effect of prolonged high pressure on cardiovascular function. Theoretically, APRV is an ideal protective mechanical ventilation strategy (Downs and Stock, 1987; Kollisch-Singule et al., 2014a; Jain et al., 2016; Zhong et al., 2020). Our previous study showed that early application of APRV could improve oxygenation and respiratory system static compliance (Cstat), reduce the MV duration, and length of intensive care unit (ICU) stay in ARDS patients (Zhou et al., 2017). Bryanna Emr et al. (2013) reported that APRV could significantly reduce histopathologic changes and bronchoalveolar lavage fluid total protein and prevent VILI in normal lungs compared with continuous mandatory ventilation, which indicated APRV might improve pulmonary permeability.

To date, there is still lack of study regarding the effects of APRV and LTV ventilation on pulmonary permeability in ARDS. We hypothesized that APRV could improve pulmonary permeability in ARDS compared with LTV ventilation. The aim of this study was to evaluate the effect of APRV and LTV ventilation on pulmonary permeability in the ARDS pig model.

## Materials and Methods

This animal experiment was approved by the Committee for the Humane Use of Animals at Sichuan University, and the project number was 2018073A. Animal experiments were performed in accordance with the Guidance for the Care and Use of Laboratory Animals (National Research Council Committee for the Update of the Guide for the C and Use of Laboratory A, 2011).

### Animal Preparation

Prior to the study, the animals were allowed to fast overnight. Ten healthy female mini Bama adult pigs (Laboratory Animal Center, Sichuan University, China) weighing 30 ± 2.5 kg were anesthetized with an intramuscular injection of ketamine hydrochloride (3 mg/kg) and atropine (2 mg/kg) and an intravenous infusion of propofol (1.0–3.0 mg/kg/h), fentanyl citrate (0.5–2.0 μg/kg/h), and midazolam (0.1–0.3 mg/kg/h) to maintain a surgical plane of anesthesia; the anesthetic drugs were titrated to achieve the target with a moderate level of autonomous breathing and no obvious pig–ventilator asynchrony. Pigs were placed in the supine position, and an electric blanket was used to maintain pigs’ body temperature at 37.3–38°C. During surgery, animals were treated with intravenous fluid resuscitation, and vasopressors were titrated to maintain the pigs’ mean arterial pressure (MAP) > 80 mmHg. Maintenance intravenous fluid requirements were calculated by body weight and given *via* continuous infusion. Balanced electrolyte solution (5–20 ml/kg/h)/normal saline (5–20 ml/kg/h) was used for maintenance and resuscitation, as needed. Ultrasound was used to determine whether there is fluid retention in animals.

Following anesthesia, tracheostomy was performed, and pigs received mechanical ventilation (PB840 Medtronic) with volume assist-controlled ventilation mode (A/C-VCV): tidal volume (Vt) 6–8 ml/kg, respiratory rate 12–16 frequency/min, FiO_2_ 40%, and positive end-expiratory pressure (PEEP) 5 cm H_2_O. Femoral arterial and central venous catheterization and gastric and urinary catheterization were performed under sterile conditions. A pulse indicator cardiac output (PiCCO) (Pulsion Medical System, Munich, Germany) monitor was used to calculate the extravascular lung water index (EVLWI) and pulmonary vascular permeability index (PVPI). Animals were continuously monitored and treated by the investigators for 48 h during the whole experiment.

### Pig Models of ARDS Establishment

Blood sample and baseline measurements of lung mechanics, hemodynamics, and arterial blood gases were collected after surgical preparation. Because of the complex pathophysiological changes of ARDS, models that could perfectly mimic human ARDS are still lacking nowadays. The saline lavage was used to establish the ARDS model in this study, which was recommended to be the good model to test ventilatory strategies (Rocco and Nieman, 2016). Under mechanical ventilation, the ARDS model was established by repeatedly perfusing at 37°C, 50 ml saline into bilateral bronchus through a fiberoptic bronchoscope (60 ml/kg saline in total). Alveolar lavage was repeated every 10 min, and negative pressure was applied to remove excessive fluid after mechanical ventilation for 3–5 min. The severe ARDS model was successfully established when the PaO_2_/FiO_2_ ratio was less than or equal to 100 mmHg and remained unchanged for 30 min (Keenan et al., 2018).

### Mechanical Ventilation

After successful establishment of pig models of severe ARDS, animals were randomized into the APRV group (*n* = 5) or the LTV (*n* = 5) group, and random sequences were generated using the standard = RAND () function in Microsoft Excel. In the APRV group, animals would be ventilated with APRV for 48 h; in the LTV group, animals would be ventilated with LTV for 48 h. During the experiments, the ventilator parameters were adjusted, according to lung mechanics and arterial blood gas analysis, to achieve the following goals: Pplat ≤ 30 cmH_2_O, PaO_2_ between 55 and 100 mmHg, PaCO_2_ between 30 and 50 mmHg, and pH ≥7.30. Investigators could not be blinded since the mechanical ventilation strategies were obviously different. But investigators were blinded to the group allocation during the sample detection and data analysis.

During 48 h mechanical ventilation, vital signs were recorded per hour, arterial blood gas analysis and the measurement of lung mechanics were performed every 4 h, and PiCCO-related indices were corrected and recorded every 6 h or when indicators such as vital signs or ventilator parameters changed. Plasma was collected at the time points just before modeling (T0) and at 0 (T1), 24 (T2), and 48 (T3) hours after modeling. Lung samples and bronchoalveolar lavage fluid (BALF) were collected at necropsy.

#### APRV Group

The mechanical ventilation mode was transitioned from A/C-VCV to APRV ventilation with the following settings: high airway pressure (P_high_) was equal to the Pplat, which was measured at VCV settings with the optimum PEEP, not exceeding 30 cmH_2_O; low airway pressure (P_low_): 5 cmH_2_O; the time spent at Plow (T_low_): initial T_low_ was set at 1- to 1.5-fold the expiratory time constant and then adjusted to achieve a termination of expiratory flow rate ≥75% of the peak expiratory flow rate (PEF); if Vt is less than 6 ml/kg and the pig–ventilator asynchrony occurred, it is allowed to gradually extend T_low_ to maintain a termination of expiratory flow rate more than 50% PEFR; release frequency: initially set at 10–14 frequency/min, and then, the release frequency (not exceeding 30 frequency/min) and depth of sedation were jointly adjusted to achieve the blood gas goal (PaO_2_ 55–100 mmHg, PaCO_2_ 30–50 mmHg, and pH ≥7.3) and the spontaneous minute ventilation goal (0%–20% of total minute mechanical ventilation); the time spent at P_high_ (T_high_) was determined by T_low_ and release frequency. A more detailed APRV parameter adjustment strategy has been described in the previous study (Zhou et al., 2017).

#### LTV Group

In the LTV group, animals received A/C-VCV ventilation with initial settings: Vt: 6 ml/kg and respiratory rate: 10–14 frequency/min; PEEP was adjusted according to the ARDSnet FiO_2_-PEEP table. Then, PEEP could be further titrated by the ways of optimum respiratory compliance. If necessary, Vt was allowed for 4–8 ml/kg to minimize asynchrony between the pig and the ventilator. Overall, the respiratory rate and tidal volume were titrated to achieve the above blood gas analysis goal and the target Pplat (not exceeding 30 ml H_2_0), according to the ARDSnet protocol. If PaO_2_:FiO_2_ ratio was less than 150 mmHg, recruitment maneuver would be considered. If the animals presented severe respiratory acidosis (pH <7.15), the respiratory rate could be increased to 35 frequency/min. A more detailed LTV parameter adjustment strategy has been described in the previous study (Zhou et al., 2017).

### Necropsy

After 48 h mechanical ventilation, pigs were euthanized. To compare the gross pathology changes between the groups, the lungs were inflated with the same airway pressure at 15 cmH_2_O. The lungs were removed, and the right lung was filled with 10% formalin for further histopathologic analysis. The left lung was sampled for analysis of the wet/dry weight ratio.

The right lung was divided into upper, middle, and lower lobes according to the anatomical structure, and then, each lobe of the lung was divided into three parts according to the dorsal and ventral lung regions. Three specimens of each part were taken that did not include mainstem airway and central vessels, and a total of nine specimens were obtained from each lobe of the lung. The method of specimen sampling was the same between the two groups.

### Quantitative Histology

The quantitative histology pathological injury score of the lung tissue was based on randomly selected photomicrographs (10 per animal). Pathologists were blinded to take samples and evaluate them. Three pathologists independently scored the photomicrograph and calculated the average score. Each photomicrograph was scored using a 3-point scale for each of five parameters: neutrophils in the alveolar space, neutrophils in the interstitial space, hyaline membranes, proteinaceous debris filling the airspaces, and alveolar septal thickening, as described in an Official American Thoracic Society Workshop Report (Matute-Bello et al., 2011).

#### Wet/Dry Weight Ratio

After necropsy, the left lung was taken out, and the surface blood was drawn. Then, the lung tissue was weighed and recorded. After that, the lung tissue was placed in the oven and dried at 80°C constant temperature. The weight was recorded every 12 h until it did not change, and the wet/dry weight ratio of lung tissue was calculated.

### BALF and Plasma

The protein contents of angiopoietin 2 (Ang-2) and vascular endothelial growth factor (VEGF) in plasma were determined using enzyme-linked immunosorbent assay (ELISA), according to the manufacturer’s recommendations at the time points just before modeling (T0) and at 0 (T1), 24 (T2), and 48 (T3) hours after modeling. BALF was harvested after 48 h mechanical ventilation with 20 ml of normal saline to determine the total protein using the bicinchoninic acid (BCA) method.

### Western Blot and Immunohistochemistry

The content changes of claudin-5, occludin, E-cadherin, and VE-cadherin proteins were determined in the lung tissues by Western blotting (WB). The right lung was divided into upper, middle, and lower lobes, according to the anatomical structure. The average value of the upper, middle, and lower lobes of lung was taken to compare the protein content in the whole lung between the groups. Lung tissues were homogenized, and 30 g of protein was electrophoresed. Membranes were exposed overnight at 4°C to the E-cadherin antibody (1:1,000; 610181; BD Transduction Laboratories™), VE-cadherin antibody (1:1,000; ab33168; Abcam), occludin antibody (1:1,000; DF7504; Affinity), and claudin-5 antibody (1:500; AF5216; Affinity). Anti-GAPDH antibody (1:1,000; BX008; BioX) was used as the loading control. Immunohistochemistry staining of E-cadherin and VE-cadherin was performed according to the manufacturer’s recommendations.

### Transmission Electron Microscopy

Transmission electron microscopy was used to evaluate the ultrastructural changes of lung tissue. The right lung was divided into upper, middle, and lower lobes, according to the anatomical structure. Lung tissues were minced into small pieces and washed with PBS and then fixed in 2.5% glutaraldehyde at 4°C overnight. The ultrastructural changes of lung tissue were examined by transmission electron microscopy (Hitachi H-7650, Hitachi, Naka, Japan).

### Statistics

All statistical analyses were performed by SPSS 23.0 (Statistical Product and Service Solutions, IBM, United States). Data are expressed as mean ± SD. The *t* tests were used to compare the differences between the groups. Repeated measures of analyses of variance with time and treatment as random effects were performed to compare differences between treatment groups for continuous parameters. Probability values less than 0.05 were considered significant.

## Results

One animal assigned to the APRV group was excluded due to the occurrence of severe anaphylactic shock during the modeling process. For the other nine pigs, ARDS was successfully induced with 2.3 ± 0.5 L bronchoalveolar lavages. The PaO_2_/FiO_2_ ratio was significantly decreased after the final lavage (66.9 ± 25.3 mmHg) compared to baseline (417.9 ± 29.39 mmHg); and CStat was significantly decreased after the final lavage (16.3 ± 4.1) compared to baseline (43.4 ± 9.4).

### Respiratory and Hemodynamic Parameters

**FIGURE 1:**
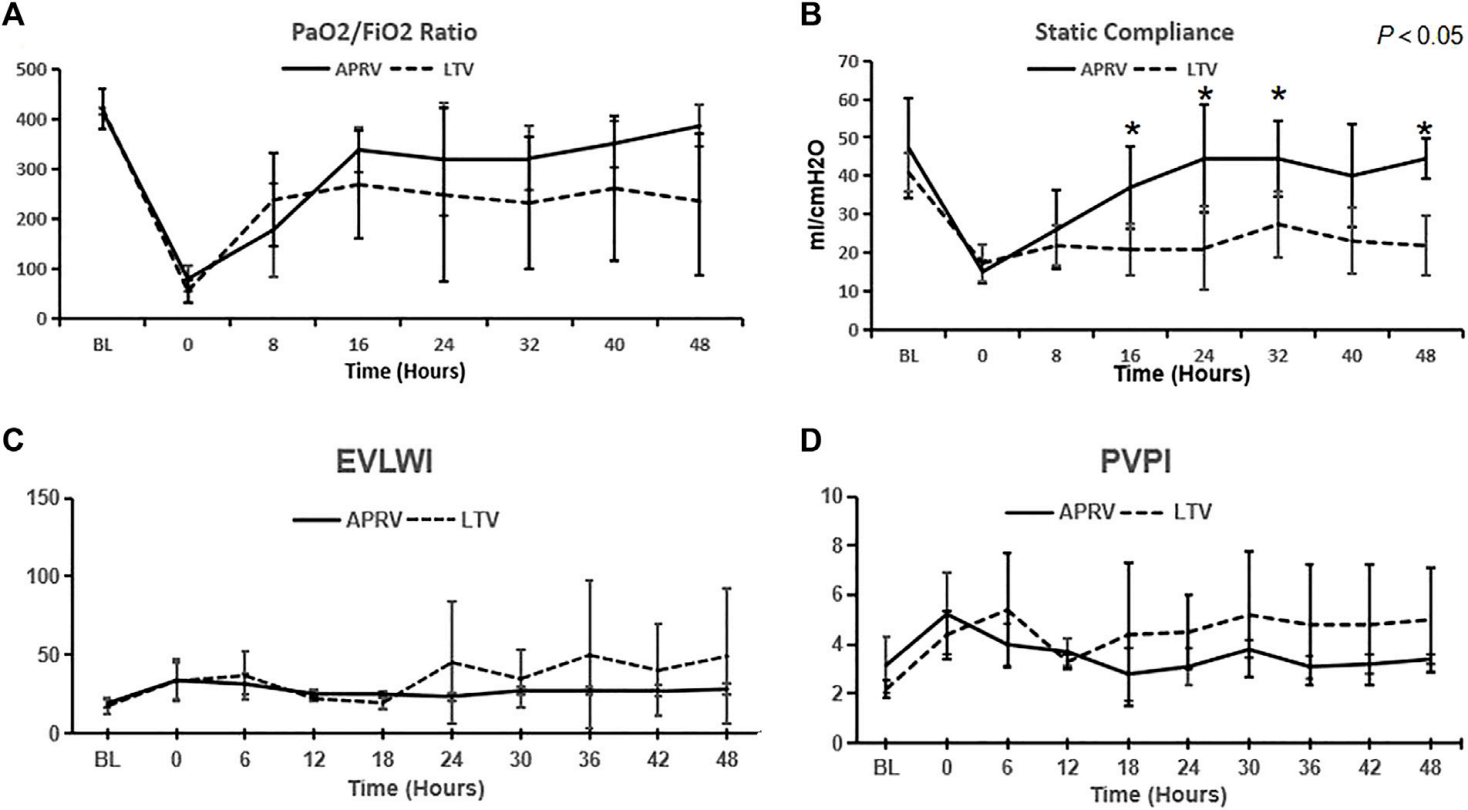
Respiratory and hemodynamic parameters. The values of **(A)** PaO_2_/FiO_2_, **(B)** Cstat, **(C)** EVLWI, and **(D)** PVPI over time were calculated. The values were expressed as the mean ± SD. *p < 0.05. APRV, airway pressure release ventilation; LTV, low tidal volume ventilation; Cstat, respiratory system static compliance; EVLWI, extravascular lung water index; PVPI, pulmonary vascular permeability index.

The results of the arterial PaO_2_/FiO_2_ ratio and CStat measurement throughout the 48-h experiment are shown in Figures 1A,B. Although the PaO_2_/FiO_2_ ratio of the APRV group was better than that of the LTV group after 16 h of mechanical ventilation, the PaO_2_/FiO_2_ ratio between the two groups showed no significant difference (*p* > 0.05). After 48 h of mechanical ventilation, although without statistically significant difference, the PaO_2_/FiO_2_ ratio of the APRV group was obviously higher than that in the LTV group (385.85 ± 42.09 vs. 235.53 ± 148.69, *p* > 0.05) (Figure 1A). The results of repeated measurement ANOVA showed that CStat in the APRV group was significantly higher than that in the LTV group (*p* < 0.05). At the time points 16, 24, 32, and 48 h, the value of CStat in the APRV group was higher (*p* < 0.05).

EVLWI and PVPI monitored by the PiCCO monitor are shown in Figures 1C,D. EVLWI and PVPI in the LTV group were higher than those in the APRV group after 24 h MV, but the difference was not statistically significant.

Respiratory parameters (including ventilator settings and monitoring parameters, pH, PaCO_2_, and PaO_2_/FiO_2_ ratio) are shown in Supplementary Table 1. Hemodynamic data including MAP, heart rate, cardiac index, vasopressor dosage, urine volume, and fluid resuscitation are shown in Supplementary Table 2. The results showed that there was no difference between the groups in the exhaled tidal volume (Vte), mean airway pressure (Pmean), pH, PaCO_2_, MAP, heart rate, cardiac index, vasopressor dosage, urine volume, and fluid resuscitation.

#### Pathological Changes of Lung Tissue

**FIGURE 2:**
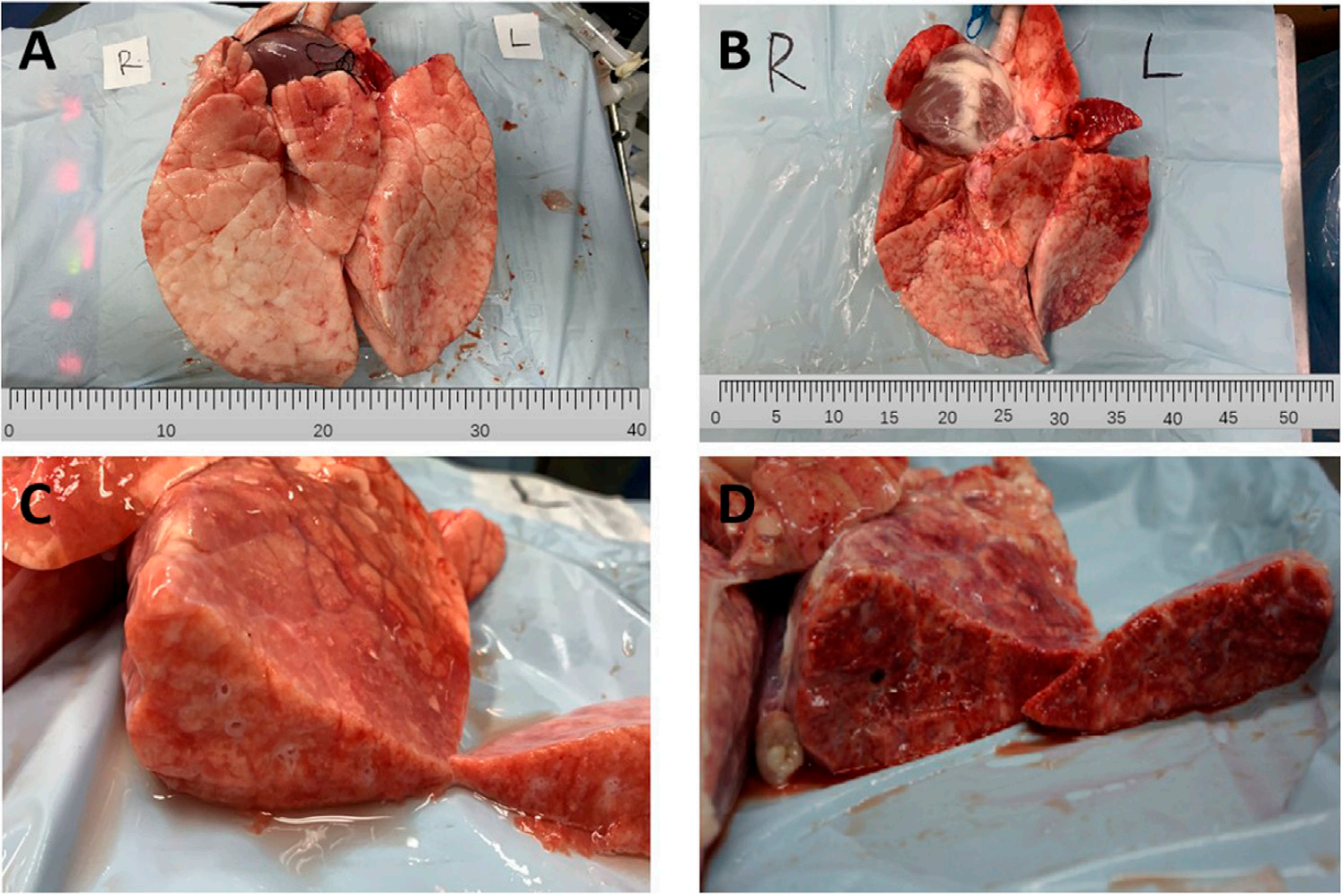
Gross pathological changes of lung tissues. Representative specimens of the gross lung form of APRV and LTV groups are shown. **(A)** APRV whole lung; **(B)** LTV whole lung; **(C)** APRV cut surface; **(D)** LTV cut surface. APRV, airway pressure release ventilation; LTV, low tidal volume ventilation.

The typical gross pathological changes of lung tissues in the APRV and LTV groups are shown in Figure 2, and there was obvious lung injury in both the APRV (Figure 2A) and the LTV groups (Figure 2B). Compared with the APRV group, inhomogeneous lung injury in the LTV group was more significant, with obvious piebald changes and lung consolidation. The cut surface of the representative LTV (Figure 2D) lung specimen shows significant pulmonary capillary occlusion and pulmonary hemorrhage compared with the APRV group (Figure 2C).

**FIGURE 3:**
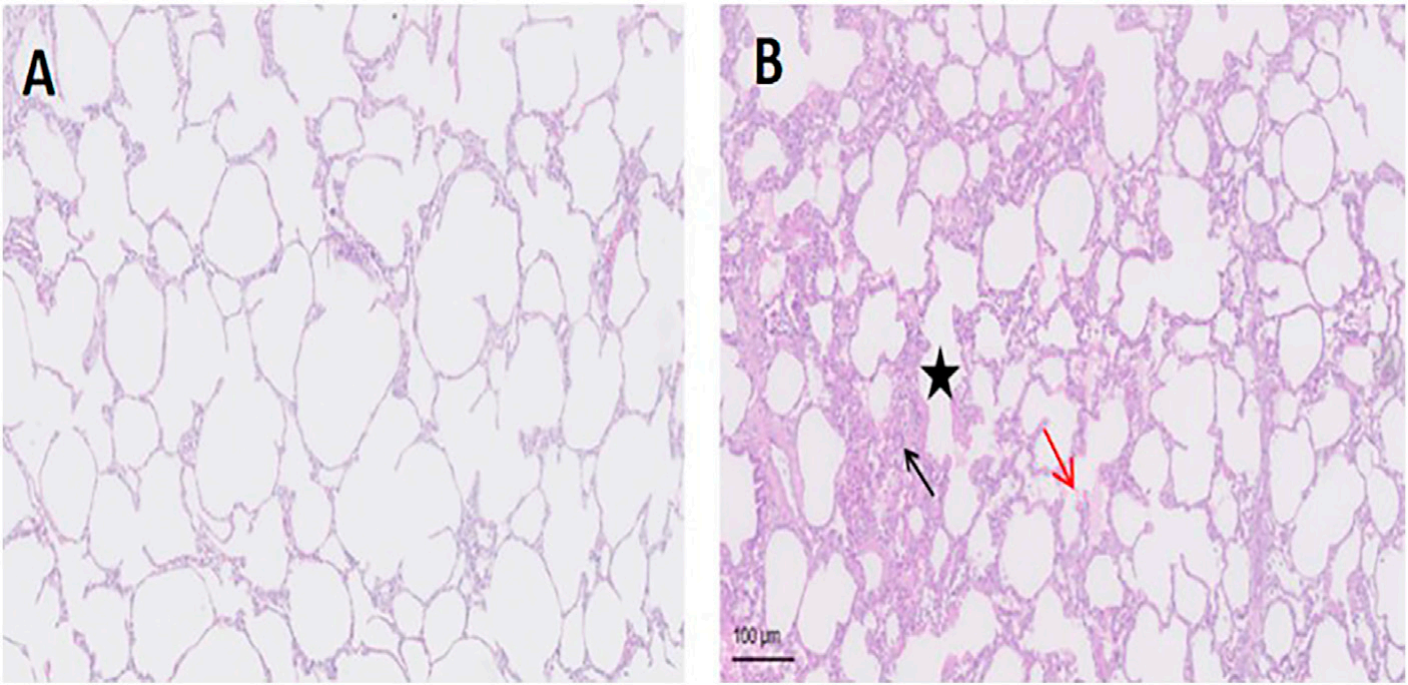
Lung histology of the representative lung histological specimen of HE staining from the LTV and APRV groups is shown. **(A)** APRV; **(B)** LTV. Black arrow: the infiltration of neutrophils in alveoli; red arrow: protein fragments in alveoli; star: thickened alveolar wall. APRV, airway pressure release ventilation; LTV, low tidal volume ventilation; HE, hematoxylin–eosin staining.

The representative lung histological specimens of the APRV (Figure 3A) and the LTV (Figure 3B) groups are shown in Figure 3. When individual injury variables were analyzed, the infiltration of neutrophils in alveolar space was more significant in the LTV group compared with the APRV group (black arrow). In the LTV group, there were obvious alveolar wall thickening (star) and significant alveolar structure change. Compared with the LTV group, the pathological injury score of lung tissue in the APRV group was lower (66.88 ± 3.01 vs. 77.5 ± 7.24; *p* < 0.05) (Table 1).

**TABLE 1:** Histological injury scores.

	APRV	LTV
Neutrophils in the alveolar space	11.25 ± 1.50*	14.00 ± 1.87
Neutrophils in the interstitial space	18.50 ± 1.29	18.40 ± 1.82
Hyaline membranes	9.75 ± 2.98	11.80 ± 3.11
Proteinaceous debris filling the airspaces	14.50 ± 1.73	17.60 ± 3.21
Alveolar septal thickening	7.50 ± 3.32*	15.80 ± 1.64
Total injury scores	66.88 ± 3.01*	77.50 ± 7.24

Values shown are means ± SD. APRV, airway pressure release ventilation; LTV, low tidal volume ventilation; *p < 0.05.

To assess the degree of pulmonary edema, we assessed the wet/dry weight ratio and the total protein in BALF after 48 h MV. There was no significant difference in the lung wet/dry weight ratio between the groups (7.86 ± 0.44 vs. 7.06 ± 0.92; *p* > 0.05) (Supplementary Figure 1A). The total protein content in BALF of the LTV group was higher than that in the APRV group, but the difference was not statistically significant (13.18 ± 7.94 vs. 18.20 ± 12.89 mg/ml; *p* > 0.05) (Supplementary Figure 1B).

### Biomarker of Pulmonary Permeability

**FIGURE 4:**
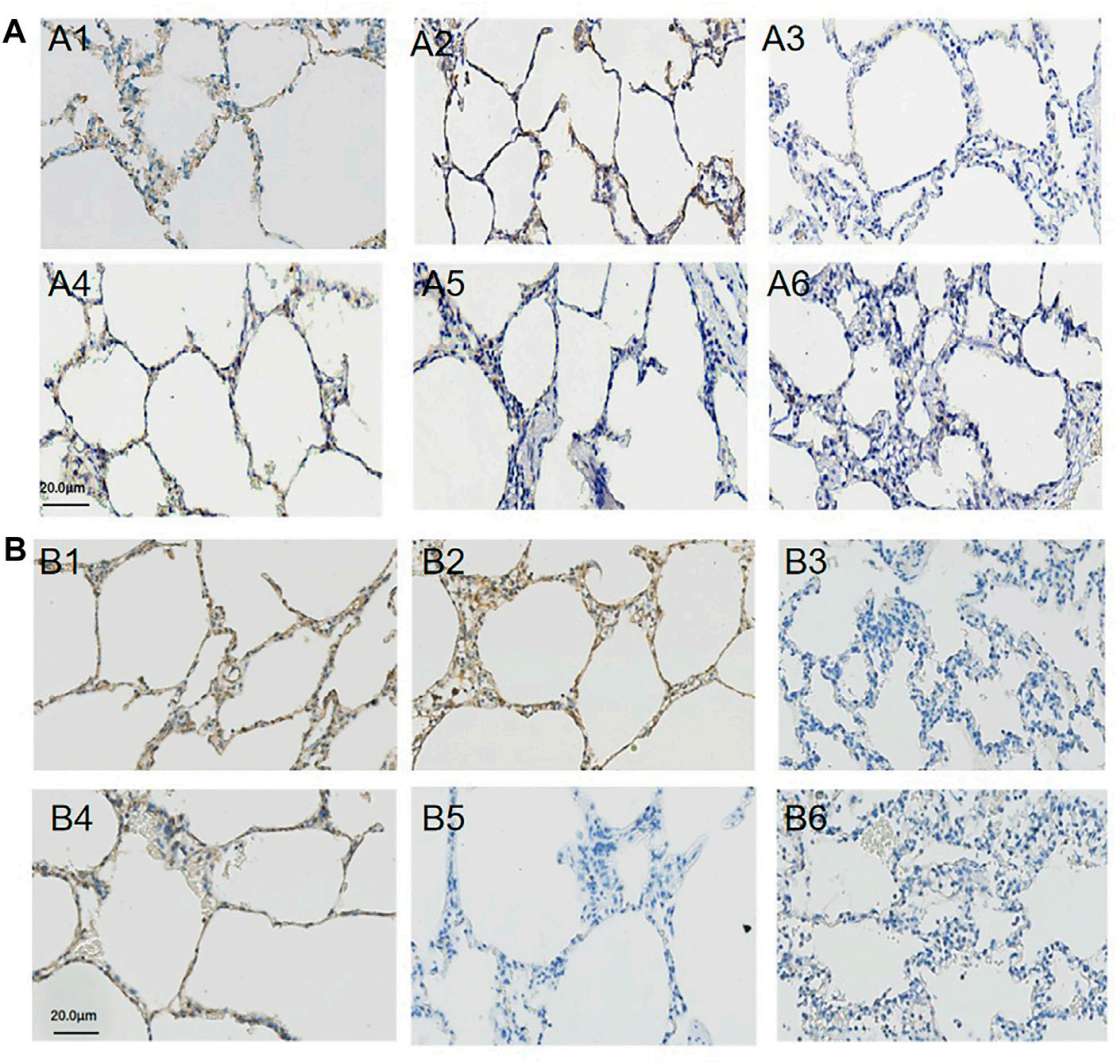
Immunohistochemistry staining of E-cadherin and VE-cadherin. The expression and distribution of E-cadherin and VE-cadherin in the right lung were analyzed by immunohistochemistry staining. E-cadherin: lung tissues were obtained from the APRV group (**A1**: the upper lobe of lung; **A2**: the middle lobe of the lung; **A3**: the lower lobe of the lung) and LTV group (**A4**: the upper lobe of the lung; **A5**: the middle lobe of the lung; **A6**: the lower lobe of the lung) after whole lung saline lavage and 48 h of mechanical ventilation. VE-cadherin: lung tissues were obtained from the APRV group (**B1**: the upper lobe of the lung; **B2**: the middle lobe of the lung; **B3**: the lower lobe of the lung) and LTV group (**B4**: the upper lobe of the lung; **B5**: the middle lobe of the lung; **B6**: the lower lobe of the lung) after whole lung saline lavage and 48 h of mechanical ventilation. Brown means positive staining. Magnification: ×400. APRV, airway pressure release ventilation; LTV, low tidal volume ventilation.

**FIGURE 5:**
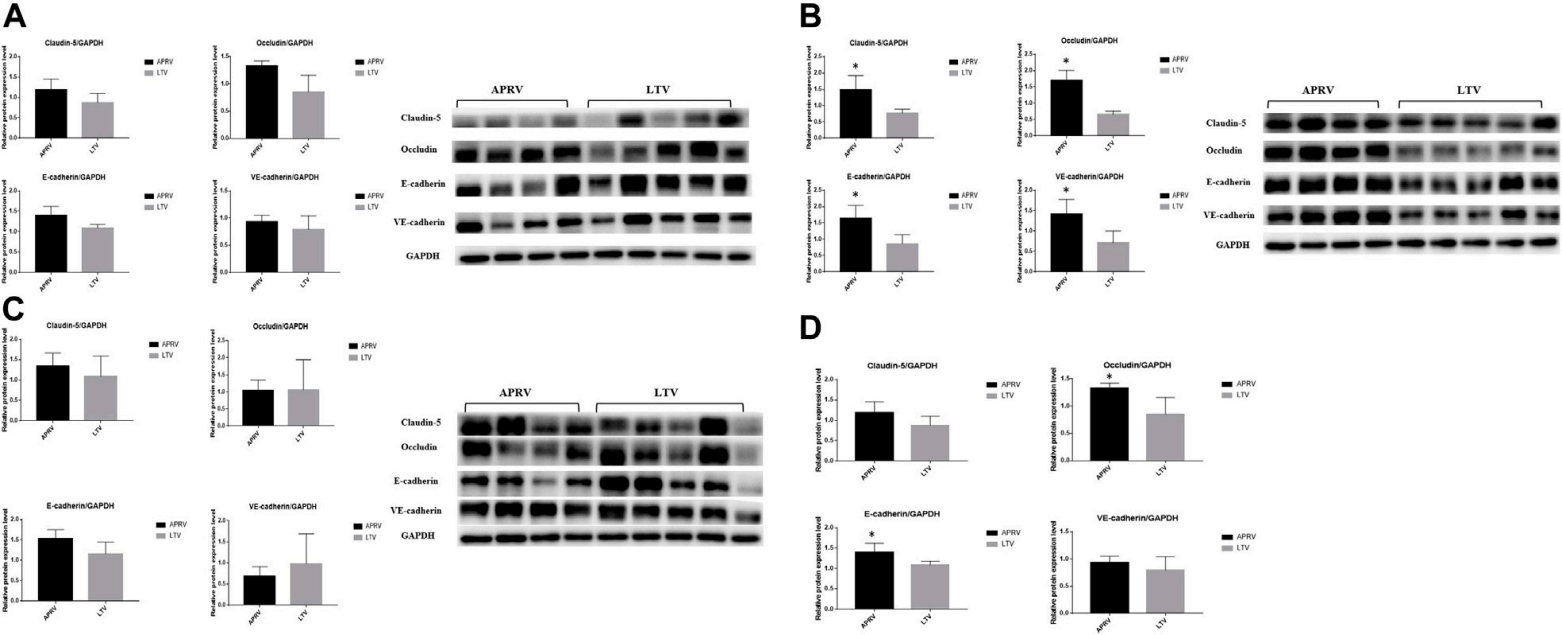
Protein expression level of biomarkers in the lung. Western blot analysis of claudin-5, occludin, E-cadherin, and VE-cadherin in the pig lung tissue. **(A)** Polyacrylamide gel electrophoresis and protein expression levels of claudin-5, occludin, E-cadherin, and VE-cadherin in the upper lobe of the right lung. **(B)** Polyacrylamide gel electrophoresis and protein expression levels of claudin-5, occludin, E-cadherin, and VE-cadherin in the middle lobe of the right lung. **(C)** Polyacrylamide gel electrophoresis and protein expression levels of claudin-5, occludin, E-cadherin, and VE-cadherin in the lower lobe of the right lung. **(D)** Protein expression levels of claudin-5, occludin, E-cadherin, and VE-cadherin in the whole right lung. The average WB results of the upper, middle, and lower lobes were taken to compare the expression of protein in the whole lung. *p < 0.05. APRV, airway pressure release ventilation; LTV, low tidal volume ventilation.

The immunohistochemistry staining of E-cadherin (Figure 4A) and VE-cadherin (Figure 4B) is shown in Figure 4, and the expressions of E-cadherin and VE-cadherin in the middle lobe of the APRV group were higher than those of the LTV group. The results of WB are shown in Figures 5A–D. Compared with LTV, the expressions of claudin-5, occludin, E-cadherin, and VE-cadherin were higher in the APRV group in the middle lobe of lungs (Figure 5B) (*p <* 0.05), and the expressions of E-cadherin and occludin were higher in the APRV group in the whole lung (Figure 5D) (*p <* 0.05).

The expression of Ang-2 in the APRV group was significantly lower than that in the LTV group after 48 h MV (19,453 ± 1,332 vs. 22,838 ± 994 pg/ml; *p <* 0.05) (Supplementary Figure 2A). There was no significant difference of the VEGF level between the APRV and LTV groups at any time point during the study (Supplementary Figure 2B).

### Transmission Electron Microscopy

**FIGURE 6:**
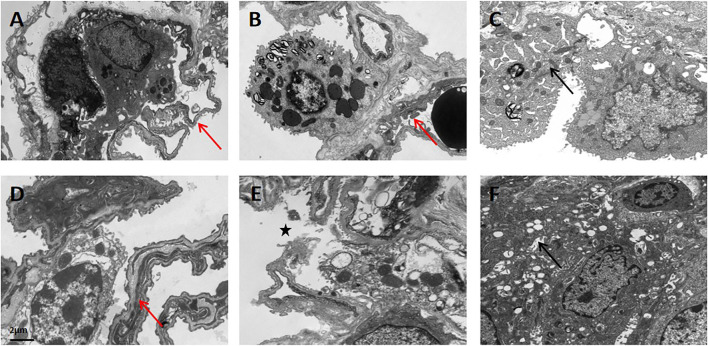
Ultrastructural changes in lung tissue in the APRV and LTV groups by transmission electron microscopy. Lung tissues were obtained from the APRV group: **(A)** the upper lobe of the lung, **(B)** the middle lobe of the lung, and **(C)** the lower lobe of the lung and the LTV group: **(D)** the upper lobe of the lung, **(E)** the middle lobe of the lung, and **(F)** the lower lobe of the lung after whole lung saline lavage and 48 h of mechanical ventilation. Magnification: ×8,000. Red arrow: tact alveolar–capillary barrier; star: destroyed alveolar–capillary barrier; black arrow: degenerated alveolar type II epithelial cells. APRV, airway pressure release ventilation; LTV, low tidal volume ventilation.

Ultrastructural changes of lung tissue in the APRV (Figures 6A–C) and LTV (Figures 6D–F) groups under transmission electron microscopy are shown in Figure 6. In the upper lobe of the right lung, the lung microvascular endothelial cells and basement membrane were intact in both the APRV (Figure 6A) and LTV (Figure 6D) groups. The structure of alveolar type II epithelial cells was preserved well, and the nucleus was obvious (Figures 6A,D). Compared with APRV, the alveolar–capillary barrier was more severely impaired in the LTV group in the middle lobe of the right lung, which was shown by pulmonary vascular endothelial cell apoptosis and basement membrane destruction, and the alveolar type II epithelial cells were degenerated and destroyed in the LTV group (Figures 6B,E). In the lower lobe of the right lung, alveolar type II epithelial cells and the alveolar–capillary barrier were severely impaired in both the APRV and LTV groups (Figures 6C,F).

## Discussion

In this experiment, an animal model of severe ARDS was successfully established by the whole lung saline lavage; animals were then randomly divided into two groups for 48 h of MV with APRV or LTV. This is the first study to systematically study the effect of APRV on the ultrastructure, histopathologic examination, the expression of cell junction protein, and physiological parameters related to the pulmonary permeability in ARDS. The results showed that compared with LTV, APRV ventilation improved CStat and the PaO_2_/FiO_2_ ratio after 48 h ventilation and was associated with a trend to decrease EVLWI and PVPI and with less serious lung histopathologic injury and alveolar–capillary barrier damage, and APRV ventilation also decreased the level of Ang-2 in the plasma and increased the content of cell junction protein in lung tissue after 48 h MV.

Increased pulmonary permeability is a typical pathophysiological change in ARDS and an important index to distinguish ARDS from cardiogenic pulmonary edema. Various factors can lead to pulmonary edema and consolidation secondary to the destruction of the alveolar–capillary barrier and then lead to respiratory failure and hypoxemia. In both classical and new hypotheses, increasing the pulmonary permeability is an important link in the occurrence and development of ARDS, and improving the pulmonary permeability is the key aspect to the treatment of ARDS (Tsukimoto et al., 19851991; Albert, 2012; Thompson et al., 2017; Wang et al., 2017). APRV was originally described in 1987 by Stock and Downs. Unlike the conventional mode, which generates the tidal volume by raising the airway pressure above the PEEP, APRV provides continuous positive airway pressure with an intermittent brief releasing pressure to produce the tidal volume while allowing spontaneous breathing. Extending the duration of high airway pressure while shortening the phase of airway pressure releasing has been associated with enhanced alveolar recruitment and stabilization, and recent animal studies have suggested that the use of APRV may relieve lung injury, improve alveolar recruitment and gas exchange, and increase homogeneity in experimental ARDS as compared to the conventional low tidal volume ventilation strategy (Jain et al., 2016).

Compared with APRV, the alveolar–capillary barrier in the LTV group was more severely damaged. The destruction of the basement membrane, degenerated alveolar type II epithelial cells, and pulmonary vascular endothelial cells in the LTV group were more serious. To the best of our knowledge, this is the first study to explore the effect of APRV on the morphology of alveolar–capillary barrier in severe ARDS compared with LTV by transmission electron microscopy. This finding indicated that APRV ventilation could facilitate protecting the integrity of the alveolar–capillary barrier, which might favor the improvement in pulmonary permeability. The results of the lung histopathological injury score and cross pathology showed that the histopathologic injury of lung was less serious in the APRV group. Both inflammatory reaction and pulmonary hemorrhage are more severe in the LTV group, which was consistent with previous studies finding that the preemptive application of APRV blocked early drivers of lung injury, preventing the occurrence of ARDS (Emr et al., 2013).

Biomarkers of pulmonary permeability in plasma and lung tissues were detected to further determine the changes in pulmonary permeability. Previous studies have found that Ang-2 is the main regulator of pulmonary vascular permeability. Increased and early changes of the Ang-2 level are independently related to the mortality of ARDS patients (van der Heijden et al., 2008; Calfee et al., 2012; Terpstra et al., 2014; Yu et al., 2021). In this study, the plasma Ang-2 protein level in the APRV group after 48 h MV was lower, suggesting that APRV has advantages in alleviating pulmonary permeability over LTV in a severe ARDS animal model. Pulmonary permeability is mainly determined by an alveolar epithelial capillary endothelial barrier. There are three main ways of intercellular connection: closed connection, anchored connection, and communication connection. E-cadherin and VE-cadherin are the major functional proteins of anchored connection, and claudin-5 and occludin are the major functional proteins of a closed connection. Studies have found that E-cadherin, VE-cadherin, claudin-5, and occludin were all affected by mechanical stretch, and their contents were directly related to lung permeability (Chiu et al., 2004; Zhao et al., 2014; Wang et al., 2017; Qiu et al., 2018). Results in our experiments showed that the expressions of E-cadherin and occludin were higher in the APRV group, which suggested that compared with LTV, the cell junctions of lung tissue were more intact and the damage of alveolar–capillary barrier was less severe in the APRV group. It is worth noting that difference in the content of cell junction-related proteins is mainly reflected in the middle lobe of the lung. Previous study showed that the middle lobe of the lung might be more vulnerable to injury during mechanical ventilation (Gattinoni and Pesenti, 2005), possibly that more recruitable lung units in the middle lobe of the lung and high shear force might generate from the cyclic opening and collapse of the atelectasis area during LTV.

After MV for 48 h, CStat in the APRV group was significantly better than in the LTV group, and the PaO_2_/FiO_2_ ratio in the APRV group was slightly higher, which were consistent with the previous animal experiments and our earlier clinical findings (Kollisch-Singule et al., 2014a; Jain et al., 2016; Zhou et al., 2017; Zhong et al., 2020). The present results also showed that in the severe ARDS animal model, APRV had a trend to reduce EVLWI and PVPI compared with LTV. Clinical studies demonstrated that the levels of EVLWI and PVPI were directly proportional to the severity of ARDS and were independent risk factors of death in patients with ARDS (Jozwiak et al., 2013; Kushimoto et al., 2013; Tagami and Ong, 2018). This finding further demonstrated that APRV might improve the pulmonary permeability of severe ARDS compared with LTV.

Previous studies have showed that APRV could reduce pulmonary edema and bronchoalveolar lavage fluid total protein in the extrapulmonary ARDS model and could prevent VILI in normal lungs (Roy et al., 2012; Emr et al., 2013). However, in our study using saline bronchoalveolar lavage to establish the pulmonary ARDS model, no significant difference was observed in the lung wet/dry weight ratio and bronchoalveolar lavage fluid total protein between the APRV and LTV groups. The differences possibly resulted from different ARDS models used in our experiment and previous studies and different ventilation strategies used for the control group. A large body of preclinical studies found that pulmonary ARDS originates in an insult to the lung epithelium and consequently leads to more severe lung epithelial injury, with heterogeneous areas of collapsed alveoli, alveolar consolidation and flooding, and inflammatory infiltration, as compared to extrapulmonary ARDS (Jabaudon et al., 2018), and animals with pulmonary ARDS seemed less responsive to higher PEEP levels and recruitment maneuver compared with extrapulmonary ARDS (Kloot et al., 2000). The study of Silva et al. (2018) found that biological response to APRV depends on ARDS etiology, and APRV ameliorated the histologic features of lung injury with more pronounced effects in extrapulmonary ARDS. Furthermore, the different ventilator settings used for the control group may exhibit different outcomes (Roy et al., 2012).

In theory, LTV can avoid over expansion of normal alveoli (non-dependent lung), thereby reducing VILI. However, studies showed that lung inhomogeneity from the aerated alveoli adjacent to collapsed or liquid-filled alveoli led to locally high stress that got magnified during tidal expansion, rather than the normal alveolus over expansion, is the main cause of VILI in ARDS patients. Thus, lowering Vt to reduce overdistension in the open lung tissue, which is surrounded by a large volume of unstable tissue, may not reduce VILI (Cereda et al., 2017; Nieman et al., 2020). From this point of view, APRV can reduce VILI and pulmonary permeability in ARDS patients through gradually opening the collapsed alveolus and stabilizing the opened alveolus through continuous positive airway pressure. In this study, there was no significant difference in the tidal volume between the two groups, and the respiratory rate of APRV group was lower than that of the LTV group, but PaCO_2_ of the APRV group was significantly lower than that of the LTV group. This finding indicated that APRV recruited a larger amount of collapse lung tissue to increase the diffusion surface area for CO_2_, and APRV could favor lung recruitment.

The methodology of APRV settings has varied widely so far; thus, the results regarding APRV studies still remain controversial (Jain et al., 2016). To a certain extent, T_low_ and P_low_ settings in our study were different from previous studies. In our study, T_low_ was initially set at 1- to 1.5-fold the expiratory time constant based on the respiratory physiology (Habashi, 2005; Daoud et al., 2012) and then titrated to achieve a termination of an expiratory flow rate ≥75% of PEF. If Vt is <6 ml/kg and the pig–ventilator dyssynchrony occurred, T_low_ could be further gradually extended (at least >50% PEFR). In previous studies, Shreyas Roy and Michaela Kollisch-Singule directly set T_low_ to achieve a termination of the expiratory flow rate ≥75% of PEF (Roy et al., 2012; Roy et al., 2013; Kollisch-Singule et al., 2014b; Kollisch-Singule et al., 2015). As in our previous clinical study, we found that in some patients with severe pulmonary ARDS, although the release frequency (not exceeding 30 frequency/min) and depth of sedation were also jointly adjusted to eliminate CO_2_ and achieve blood gas target, if T_low_ was simply adjusted to achieve a termination of an expiratory flow rate ≥75% of PEF, the release tidal volume would be very low, patients would exhibit dyspnea and asynchrony, and severe hypercapnia would also be frequently encountered. Therefore, we individually titrated T_low_ to be at the target PEFR and to optimize the pig–ventilator interaction, thus reducing the use of deep sedation and neuromuscular blocking agents. In the present experiment, APRV ventilated the severe pulmonary ARDS model with the PaO_2_/FiO_2_ ratio <100 mmHg; we implemented the method of APRV settings referring our previous clinical research experience and validated its effects on this severe pulmonary ARDS model (Zhou et al., 2017). Although T_low_ was individually set, T_low_ in the present experiment was at 0.3–0.4 s, which was similar to the previous studies (Roy et al., 2013; Kollisch-Singule et al., 2014b; Kollisch-Singule et al., 2015). P_low_ was set at 5 cmH_2_O rather than 0 cmH_2_O, which was commonly used in previous studies. If P_low_ was set at 0 cmH_2_O, avoiding lung collapse would only depend on the auto-PEEP generated by shortening pressure release time. However, considering that auto-PEEP may be influenced by many factors such as respiratory system compliance, airway resistance, and active expiratory, and it may vary widely, thus worrying that might be an unreliable way to prevent alveolar collapse in some ARDS patients, we chose a low PEEP level (5 cmH_2_O) similar to the physiology transpulmonary pressure based on respiratory physiology and our previous clinical research experience and results (Daoud et al., 2012; Zhou et al., 2017). In addition, the result in this animal experiment showed that PaCO_2_ in the APRV group was lower than that in the LTV group. Regarding APRV settings, we will further study and explore it in the future.

There are several limitations to this study. First, because of the limited number of animals, oxygenation index and total protein in BALF only showed a trend to be improved in the APRV group without statistically significant difference; however, APRV has shown obvious advantages in improving pulmonary permeability in severe ARDS compared with LTV in terms of protein and histological structure. Second, animals in our experiment were mechanically ventilated with APRV or LTV for 48 h. Theoretically, the longer the mechanical ventilation, the better will be the mimic disease process, and 48 h seem to be short. But we have tried our best to extend the experiment duration for 48 h. Third, there were only the APRV group and the LTV group in this study, adding a control group that without mechanical ventilation might better prove the results. But for severe ARDS pigs, vital signs could not be maintained without mechanical ventilation. Last, this experiment found that APRV has advantages in improving the pulmonary permeability of a severe ARDS animal model, but this study only focused on the effects of APRV and LTV on the respiratory physiological function and pathophysiological findings in the severe ARDS big animal model. We will further study the potential mechanism in the future.

## Conclusion

In comparison with LTV, APRV could preserve the alveolar–capillary barrier architecture, mitigate lung histopathologic injury, increase the expression of cell junction protein, improve respiratory system compliance, and showed a trend to reduce extravascular lung water and improve oxygenation. These findings indicated that APRV might led to more profound beneficial effects on the integrity of the alveolar–capillary barrier architecture and on the expression of biomarkers related to pulmonary permeability.

## Data Availability

The raw data supporting the conclusions of this article will be made available by the authors, without undue reservation.

## Supplementary Material

Supplementary Figure S1 Wet/dry weight ratio of lung tissue and total protein in BALF. The wet/dry weight ratio of lung tissue of the APRV and LTV groups was determined **(A)**. Total protein in BALF was analyzed by the BCA method **(B)**. Left lung tissues and the BALF sample were obtained after whole lung saline lavage and 48 h of mechanical ventilation. The values were expressed as the mean ± SD. **p* < 0.05. APRV, airway pressure release ventilation; LTV, low tidal volume ventilation; BALF, bronchoalveolar lavage fluid; BCA, bicinchoninic acid.

Supplementary Figure S2 Concentrations of Ang-2 and VEGF protein in the plasma. The concentrations of Ang-2 and VEGF protein in the plasma were analyzed by ELISA. **(A)** Concentration of Ang-2 over time. **(B)** Concentration of VEGF over time. Plasma samples were obtained at baseline (BL), the time point of successful modeling (T0), after 24 h of mechanical ventilation (T24) and after 48 h of mechanical ventilation (T48). The values were expressed as the mean ± SDs. **p* < 0.05. APRV, airway pressure release ventilation; LTV, low tidal volume ventilation; Ang-2, angiopoietin 2; VEGF, vascular endothelial growth factor; ELISA, enzyme-linked immunosorbent assay.

Supplementary Table S1 Pulmonary data of APRV and LTV groups over time.

Supplementary Table S2 Hemodynamic data of APRV and LTV groups over time.

## Abbreviations

ALI, acute lung injury; Ang-2, angiopoietin 2; APRV, airway pressure release ventilation; ARDS, acute respiratory distress syndrome; BALF, bronchoalveolar lavage fluid; BCA, bicinchoninic acid; Cstat, respiratory system static compliance; ELISA, enzyme-linked immunosorbent assay; EVLWI, extravascular lung water index; HE, hematoxylin–eosin staining; LIP, low inflection point; LTV, low tidal volume; MV, mechanical ventilation; PEEP, positive end-expiratory pressure; PEF, peak expiratory flow; P_high_, pressure during inspiration/CPAP phase; PiCCO, pulse-induced contour cardiac output; P_low_, pressure during expiration/release phase; Pmean, mean airway pressure; PVPI, pulmonary vascular permeability index; T_high_, the time spent at P_high_; T_low_, the time spent at P_low_; VEGF, vascular endothelial growth factor; VILI, ventilator-induced lung injury; Vt, tidal volume; Vte, exhaled tidal volume.

## References

[B1] Albert R. K. (2012). The Role of Ventilation-Induced Surfactant Dysfunction and Atelectasis in Causing Acute Respiratory Distress Syndrome. Am. J. Respir. Crit. Care Med. 185 (7), 702–708. 10.1164/rccm.201109-1667pp 22227381

[B2] Bates J. H. T. Smith B. J. (2018). Ventilator-induced Lung Injury and Lung Mechanics. Ann. Transl. Med. 6 (19), 378. 10.21037/atm.2018.06.29 30460252 PMC6212358

[B3] Bellani G. Laffey J. G. Pham T. Fan E. Brochard L. Esteban A. (2016). Epidemiology, Patterns of Care, and Mortality for Patients with Acute Respiratory Distress Syndrome in Intensive Care Units in 50 Countries. Jama 315 (8), 788–800. 10.1001/jama.2016.0291 26903337

[B4] Calfee C. S. Gallagher D. Abbott J. Thompson B. T. Matthay M. A. (2012). Plasma Angiopoietin-2 in Clinical Acute Lung Injury. Crit. care Med. 40 (6), 1731–1737. 10.1097/ccm.0b013e3182451c87 22610178 PMC3601049

[B5] Cereda M. Xin Y. Hamedani H. Bellani G. Kadlecek S. Clapp J. (2017). Tidal Changes on CT and Progression of ARDS. Thorax 72 (11), 981–989. 10.1136/thoraxjnl-2016-209833 28634220 PMC5738538

[B6] Chiu Y.-J. Kusano K.-i. Thomas T. N. Fujiwara K. (2004). Endothelial Cell-Cell Adhesion and Mechanosignal Transduction. Endothelium 11 (1), 59–73. 10.1080/10623320490432489 15203879

[B7] Daoud E. G. Farag H. L. Chatburn R. L. (2012). Airway Pressure Release Ventilation: what Do We Know? Respir. Care 57 (2), 282–292. 10.4187/respcare.01238 21762559

[B8] Downs J. B. Stock M. C. (1987). Airway Pressure Release Ventilation: a New Concept in Ventilatory Support. Crit. Care Med. 15 (5), 459–461. 3568710

[B9] Emr B. Gatto L. A. Roy S. Satalin J. Ghosh A. Snyder K. (2013). Airway Pressure Release Ventilation Prevents Ventilator-Induced Lung Injury in Normal Lungs. JAMA Surg. 148 (11), 1005–1012. 10.1001/jamasurg.2013.3746 24026214

[B10] Fan E. Del Sorbo L. Goligher E. C. Hodgson C. L. Munshi L. Walkey A. J. (2017). An Official American Thoracic Society/European Society of Intensive Care Medicine/Society of Critical Care Medicine Clinical Practice Guideline: Mechanical Ventilation in Adult Patients with Acute Respiratory Distress Syndrome. Am. J. Respir. Crit. Care Med. 195 (9), 1253–1263. 10.1164/rccm.201703-0548ST 28459336

[B11] Fan E. Brodie D. Slutsky A. S. (2018). Acute Respiratory Distress Syndrome. Jama 319 (7), 698–710. 10.1001/jama.2017.21907 29466596

[B12] Gattinoni L. Pesenti A. (2005). The Concept of "baby Lung". Intensive Care Med. 31 (6), 776–784. 10.1007/s00134-005-2627-z 15812622

[B13] Habashi N. M. (2005). Other Approaches to Open-Lung Ventilation: Airway Pressure Release Ventilation. Crit. Care Med. 33 (3 Suppl. l), S228–S240. 10.1097/01.ccm.0000155920.11893.37 15753733

[B14] Jabaudon M. Blondonnet R. Constantin J.-M. (2018). Distinct Biological Effects of Time-Controlled Adaptive Ventilation in Pulmonary and Extrapulmonary Acute Respiratory Distress Syndrome. Crit. care Med. 46 (6), 1038–1040. 10.1097/ccm.0000000000003106 29762415

[B15] Jain S. V. Kollisch-Singule M. Sadowitz B. Dombert L. Satalin J. Andrews P. (2016). The 30-year Evolution of Airway Pressure Release Ventilation (APRV). ICMx 4 (1), 11. 10.1186/s40635-016-0085-2 27207149 PMC4875584

[B16] Jozwiak M. Silva S. Persichini R. Anguel N. Osman D. Richard C. (2013). Extravascular Lung Water Is an Independent Prognostic Factor in Patients with Acute Respiratory Distress Syndrome*. Crit. care Med. 41 (2), 472–480. 10.1097/ccm.0b013e31826ab377 23263578

[B17] Keenan J. C. Cortes-Puentes G. A. Zhang L. Adams A. B. Dries D. J. Marini J. J. (2018). PEEP Titration: the Effect of Prone Position and Abdominal Pressure in an ARDS Model. ICMx 6 (1), 3. 10.1186/s40635-018-0170-9 29380160 PMC5789120

[B18] Kloot T. E. V. d. Blanch L. Melynne Youngblood A. Weinert C. Adams A. B. Marini J. J. (2000). Recruitment Maneuvers in Three Experimental Models of Acute Lung Injury. Am. J. Respir. Crit. Care Med. 161 (5), 1485–1494. 10.1164/ajrccm.161.5.9809014 10806143

[B19] Kollisch-Singule M. Emr B. Jain S. V. Andrews P. Satalin J. Liu J. (2015). The Effects of Airway Pressure Release Ventilation on Respiratory Mechanics in Extrapulmonary Lung Injury. ICMx 3 (1), 35. 10.1186/s40635-015-0071-0 PMC4688284 26694915

[B20] Kollisch-Singule M. Emr B. Smith B. Roy S. Jain S. Satalin J. (2014). Mechanical Breath Profile of Airway Pressure Release Ventilation. JAMA Surg. 149 (11), 1138–1145. 10.1001/jamasurg.2014.1829 25230047

[B21] Kollisch-Singule M. Emr B. Smith B. Ruiz C. Roy S. Meng Q. (2014). Airway Pressure Release Ventilation Reduces Conducting Airway Micro-strain in Lung Injury. J. Am. Coll. Surg. 219 (5), 968–976. 10.1016/j.jamcollsurg.2014.09.011 25440027 PMC4350231

[B22] Kollisch-Singule M. Jain S. Andrews P. Smith B. J. Hamlington-Smith K. L. Roy S. (2016). Effect of Airway Pressure Release Ventilation on Dynamic Alveolar Heterogeneity. JAMA Surg. 151 (1), 64–72. 10.1001/jamasurg.2015.2683 26444302

[B23] Kushimoto S. Endo T. Yamanouchi S. Sakamoto T. Ishikura H. Kitazawa Y. (2013). Relationship between Extravascular Lung Water and Severity Categories of Acute Respiratory Distress Syndrome by the Berlin Definition. Crit. Care 17 (4), R132. 10.1186/cc12811 23844662 PMC4056600

[B24] Matute-Bello G. Downey G. Moore B. B. Groshong S. D. Matthay M. A. Slutsky A. S. (2011). An Official American Thoracic Society Workshop Report: Features and Measurements of Experimental Acute Lung Injury in Animals. Am. J. Respir. Cell Mol. Biol. 44 (5), 725–738. 10.1165/rcmb.2009-0210st 21531958 PMC7328339

[B25] National Research Council Committee for the Update of the Guide for the C, Use of Laboratory A (2011). The National Academies Collection: Reports Funded by National Institutes of Health. National Academy of Sciences. Washington (DC): National Academies Press (US) Copyright © 2011. Guide for the Care and Use of Laboratory Animals.

[B26] Nieman G. F. Al-Khalisy H. Kollisch-Singule M. Satalin J. Blair S. Trikha G. (2020). A Physiologically Informed Strategy to Effectively Open, Stabilize, and Protect the Acutely Injured Lung. Front. Physiol. 11, 227. 10.3389/fphys.2020.00227 32265734 PMC7096584

[B27] Qiu J.-L. Song B.-L. Wang Y.-J. Zhang F.-T. Wang Y.-L. (2018). Role of Glutamine in the Mediation of E-Cadherin, P120-Catenin and Inflammation in Ventilator-Induced Lung Injury. Chin. Med. J. 131 (7), 804–812. 10.4103/0366-6999.228230 29578124 PMC5887739

[B28] Rocco P. R. M. Nieman G. F. (2016). ARDS: what Experimental Models Have Taught Us. Intensive Care Med. 42 (5), 806–810. 10.1007/s00134-016-4268-9 26928038

[B29] Roy S. Habashi N. Sadowitz B. Andrews P. Ge L. Wang G. (2013). Early Airway Pressure Release Ventilation Prevents ARDS-A Novel Preventive Approach to Lung Injury. Shock (Augusta, Ga) 39 (1), 28–38. 10.1097/shk.0b013e31827b47bb PMC3539171 23247119

[B30] Roy S. Sadowitz B. Andrews P. Gatto L. A. Marx W. Ge L. (2012). Early Stabilizing Alveolar Ventilation Prevents Acute Respiratory Distress Syndrome. J. trauma acute care Surg. 73 (2), 391–400. 10.1097/ta.0b013e31825c7a82 22846945 PMC3521044

[B31] Silva P. L. Cruz F. F. Samary C. d. S. Moraes L. de Magalhães R. F. Fernandes M. V. d. S. (2018). Biological Response to Time-Controlled Adaptive Ventilation Depends on Acute Respiratory Distress Syndrome Etiology*. Crit. care Med. 46 (6), e609–e617. 10.1097/ccm.0000000000003078 29485489

[B32] Slutsky A. S. Ranieri V. M. (2013). Ventilator-induced Lung Injury. N. Engl. J. Med. 369 (22), 2126–2136. 10.1056/nejmra1208707 24283226

[B33] Tagami T. Ong M. E. H. (2018). Extravascular Lung Water Measurements in Acute Respiratory Distress Syndrome. Curr. Opin. Crit. care 24 (3), 209–215. 10.1097/mcc.0000000000000503 29608455 PMC6037282

[B34] Terpstra M. L. Aman J. van Nieuw Amerongen G. P. Groeneveld A. B. J. (2014). Plasma Biomarkers for Acute Respiratory Distress Syndrome. Crit. care Med. 42 (3), 691–700. 10.1097/01.ccm.0000435669.60811.24 24158164

[B35] Terragni P. P. Rosboch G. Tealdi A. Corno E. Menaldo E. Davini O. (2007). Tidal Hyperinflation during Low Tidal Volume Ventilation in Acute Respiratory Distress Syndrome. Am. J. Respir. Crit. Care Med. 175 (2), 160–166. 10.1164/rccm.200607-915oc 17038660

[B36] Thompson B. T. Chambers R. C. Liu K. D. (2017). Acute Respiratory Distress Syndrome. N. Engl. J. Med. 377 (6), 562–572. 10.1056/nejmra1608077 28792873

[B37] Tsukimoto K. Mathieu-Costello O. Prediletto R. Elliott A. R. West J. B. (19851991). Ultrastructural Appearances of Pulmonary Capillaries at High Transmural Pressures. J. Appl. Physiol. (1985) 71 (2), 573–582. 10.1152/jappl.1991.71.2.573 1718936

[B38] van der Heijden M. van Nieuw Amerongen G. P. Koolwijk P. van Hinsbergh V. W. M. Groeneveld A. B. J. (2008). Angiopoietin-2, Permeability Oedema, Occurrence and Severity of ALI/ARDS in Septic and Non-septic Critically Ill Patients. Thorax 63 (10), 903–909. 10.1136/thx.2007.087387 18559364

[B39] Villar J. Blanco J. Kacmarek R. M. (2016). Current Incidence and Outcome of the Acute Respiratory Distress Syndrome. Curr. Opin. Crit. care 22 (1), 1–6. 10.1097/mcc.0000000000000266 26645551

[B40] Wang T. Gross C. Desai A. A. Zemskov E. Wu X. Garcia A. N. (2017). Endothelial Cell Signaling and Ventilator-Induced Lung Injury: Molecular Mechanisms, Genomic Analyses, and Therapeutic Targets. Am. J. Physiology-Lung Cell. Mol. Physiology 312 (4), L452–l476. 10.1152/ajplung.00231.2016 PMC5407098 27979857

[B41] Yu W.-K. McNeil J. B. Wickersham N. E. Shaver C. M. Bastarache J. A. Ware L. B. (2021). Angiopoietin-2 Outperforms Other Endothelial Biomarkers Associated with Severe Acute Kidney Injury in Patients with Severe Sepsis and Respiratory Failure. Crit. Care 25 (1), 48. 10.1186/s13054-021-03474-z 33541396 PMC7859898

[B42] Zhao T. Liu M. Gu C. Wang X. Wang Y. (2014). Activation of C-Src Tyrosine Kinase Mediated the Degradation of Occludin in Ventilator-Induced Lung Injury. Respir. Res. 15 (1), 158. 10.1186/s12931-014-0158-2 25471013 PMC4262993

[B43] Zhong X. Wu Q. Yang H. Dong W. Wang B. Zhang Z. (2020). Airway Pressure Release Ventilation versus Low Tidal Volume Ventilation for Patients with Acute Respiratory Distress Syndrome/acute Lung Injury: a Meta-Analysis of Randomized Clinical Trials. Ann. Transl. Med. 8 (24), 1641. 10.21037/atm-20-6917 33490153 PMC7812231

[B44] Zhou Y. Jin X. Lv Y. Wang P. Yang Y. Liang G. (2017). Early Application of Airway Pressure Release Ventilation May Reduce the Duration of Mechanical Ventilation in Acute Respiratory Distress Syndrome. Intensive Care Med. 43 (11), 1648–1659. 10.1007/s00134-017-4912-z 28936695 PMC5633625

