# Supplementary figures and images for: The Effects of Airway Pressure Release Ventilation on Pulmonary Permeability in Severe Acute Respiratory Distress Syndrome Pig Models
Source: Front Physiol. 2022 Jul 22;13:927507. doi: 10.3389/fphys.2022.927507 (PMC9354663; doi:10.3389/fphys.2022.927507)

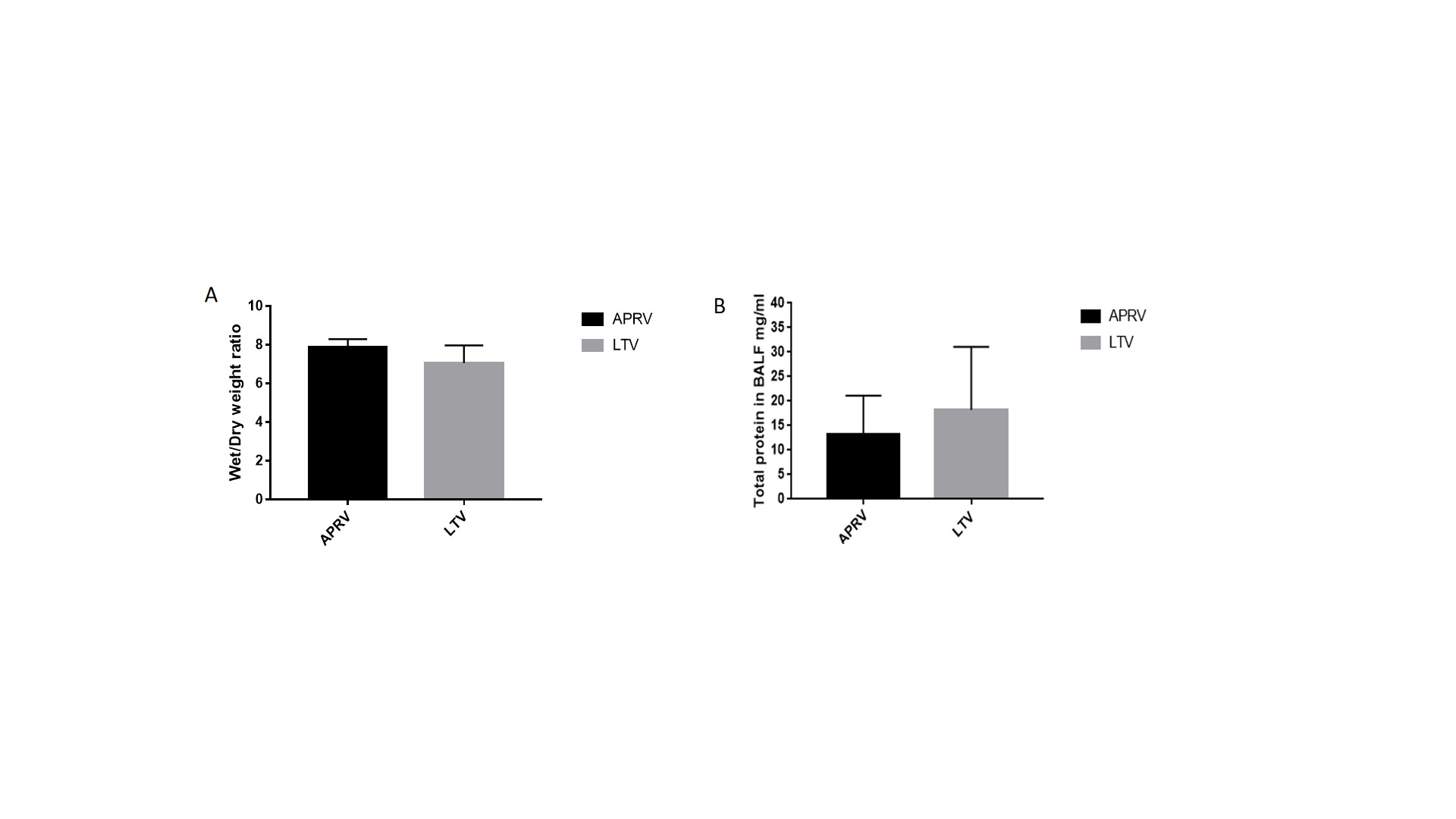

Supplement: Supplementary file 1 [file Image1.JPEG]

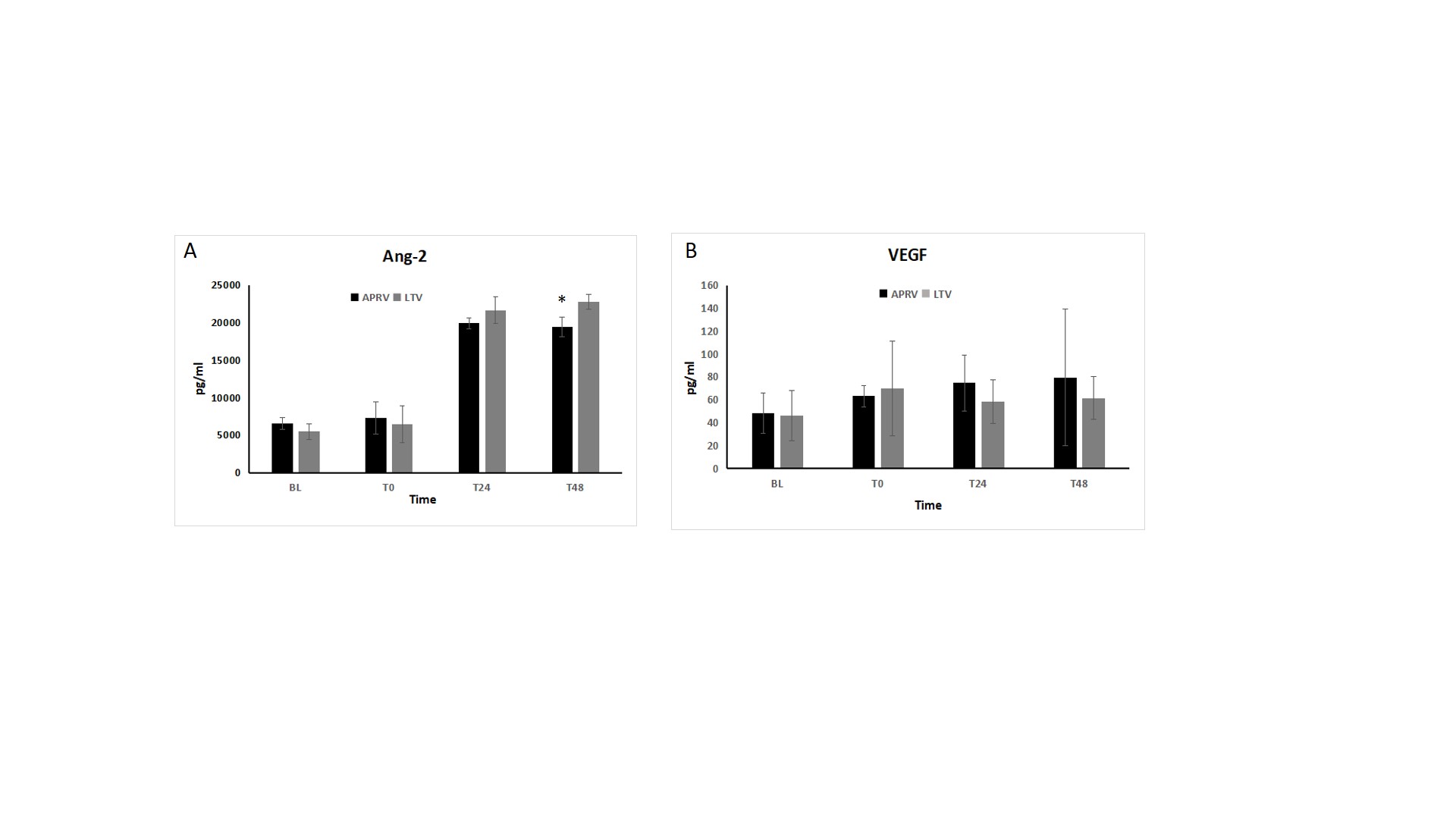

Supplement: Supplementary file 2 [file Image2.JPEG]
