# Supplementary material for: The Effects of Airway Pressure Release Ventilation on Pulmonary Permeability in Severe Acute Respiratory Distress Syndrome Pig Models
Source: Front Physiol. 2022 Jul 22;13:927507. doi: 10.3389/fphys.2022.927507 (PMC9354663; doi:10.3389/fphys.2022.927507)
Supplement: Supplementary file 3 [file DataSheet1.docx]

**Additional table 1. Pulmonary data of APRV and LTV groups over time**

|  | **Group** | **BL** | **0h** | **8h** | **16h** | **24h** | **32h** | **40h** | **48h** | **P** |
| --- | --- | --- | --- | --- | --- | --- | --- | --- | --- | --- |
| **P_high_(cmH_2_0)** | APRV | / | 25.8±0.5 | 26.8±1.5 | 25.3±1.7 | 25.5±1.3 | 24.8±2.2 | 24.0±1.6 | 24.0±1.6 | / |
| **P_low_(s)** | APRV | / | 5.0±0.0 | 5.0±0.0 | 5.0±0.0 | 5.0±0.0 | 5.0±0.0 | 5.0±0.0 | 5.0±0.0 | / |
| **Release frequency** | APRV | / | 21.0±2.0 | 21.8±6.2 | 19.8±3.3 | 17.3±3.6 | 18.0±5.0 | 17.3±3.6 | 17.3±3.6 | / |
| **T_low_(s)** | APRV | / | 0.34±0.04 | 0.33±0.04 | 0.33±0.03 | 0.33±0.03 | 0.33±0.03 | 0.33±0.03 | 0.30±0.08 | / |
| **Vt** | LTV | 7.7±0.5 | 6.9±0.9 | 7.5±0.4 | 7.3±0.5 | 7.1±0.4 | 7.1±0.4 | 7.1±0.4 | 7.1±0.4 | / |
| **RR** | LTV | 21.0±2.6 | 24.6±3.8 | 27.4±2.8 | 28.6±4.0 | 30.2±3.2 | 28.6±5.6 | 26.2±6.3 | 27.6±5.6 | / |
| **PEEP(cmH_2_0)** | LTV | 5.0±0.0 | 12.4±0.9 | 14.8±1.8 | 13.6±1.7 | 13.6±1.7 | 13.6±2.2 | 13.6±2.2 | 12.8±3.3 | / |
| **Static compliance** | APRV | 47.3±13.0 | 15.0±2.9 | 26.0±10.4 | 37.0±10.6* | 44.5±14.2* | 44.5±10.0* | 40.0±13.5 | 44.5±5.3* | # |
|  | LTV | 40.8±5.07 | 17.3±5.0 | 21.8±5.4 | 20.8±6.7 | 21.1±11.0 | 27.4±8.7 | 23.0±8.6 | 21.8±7.9 |  |
| **Vte(ml/kg)** | APRV | 6.9±0.6 | 7.0±0.8 | 6.9±1.0 | 7.3±1.3 | 7.6±1.0 | 7.0±1.4 | 7.1±1.1 | 7.3±0.9 |  |
|  | LTV | 7.7±0.5 | 7.0±0.9 | 7.5±0.4 | 7.3±0.5 | 7.1±0.4 | 7.1±0.4 | 7.1±0.4 | 7.1±0.4 |  |
| **Pmean(cmH_2_0)** | APRV | 7.4±2.8 | 14.5±4.0 | 24.8±1.0* | 23.8±1.9 | 23.0±1.6 | 21.8±1.9 | 22.0±2.7 | 23.5±1.7 |  |
|  | LTV | 7.6±1.6 | 16.0±3.8 | 19.4±3.0 | 18.6±2.7 | 19.4±3.0 | 19.0±3.4 | 18.8±3.7 | 19.6±4.8 |  |
| **PH** | APRV | 7.41±0.01 | 7.36±0.08 | 7.37±0.08 | 7.58±0.08 | 7.55±0.11 | 7.48±0.10 | 7.52±0.05 | 7.50±0.04 |  |
|  | LTV | 7.43±0.07 | 7.40±0.06 | 7.42±0.04 | 7.41±0.13 | 7.40±0.15 | 7.42±0.11 | 7.44±0.08 | 7.42±0.08 |  |
| **PaCO_2_(mmHg)** | APRV | 49.4±4.4 | 51.0±10.0 | 55.5±14.3 | 35.4±3.1* | 39.6±14.5 | 46.0±18.8 | 37.7±3.7 | 38.2±5.6* |  |
|  | LTV | 42.9±6.4 | 41.7±9.4 | 48.3±8.5 | 52.1±12.9 | 56.0±16.8 | 52.3±12.8 | 61.9±22.4 | 54.0±11.7 |  |
| **PaO_2_/FiO_2_**  **(mmHg)** | APRV | 415.2±7.6 | 80.0±25.3 | 177.8±92.8 | 337.7±44.7 | 318.8±111.0 | 321.0±64.9 | 350.6±46.8 | 385.9±42.1 |  |
|  | LTV | 420.0±40.9 | 56.3±22.2 | 237.8±93.0 | 268.5±108.7 | 247.9±172.6 | 231.9±130.3 | 261.1±145.4 | 235.5±148.7 |  |

*#* p<0.05 between groups with repeated measures analysis of variance

***p<0.05 between groups with t-test.

APRV, airway pressure release ventilation; LTV, low tidal volume; P_high_, pressure during inspiration/CPAP phase; P_low_, pressure during expiration/release phase; T_low_, the time spent at P_low_; Vt,tidal volume; RR: respiratory rate; PEEP, positive end expiratory pressure; Vte, exhaled tidal volume; Pmean, mean airway pressure; PaCO_2_, partial pressure of carbon dioxide in arterial blood; PaO_2_/FiO_2_, partial pressure of oxygen in arterial blood/fraction of inspired oxygen;

**Additional table 2. Hemodynamic data of APRV and LTV groups over time**

|  | **Group** | **BL** | **0h** | **8h** | **16h** | **24h** | **32h** | **40h** | **48h** | **P** |
| --- | --- | --- | --- | --- | --- | --- | --- | --- | --- | --- |
| **MAP (mmHg)** | APRV | 105.0±35.2 | 97.0±18.1 | 99.0±17.8 | 88.3±10.7 | 80.0±4.2 | 89.5±8.2 | 85.0±6.5 | 103.8±25.5 |  |
|  | LTV | 110.0±14.5 | 94.0±15.7 | 94.0±21.0 | 86.6±19.2 | 91.2±19.0 | 89.0±18.2 | 119.4±50.5 | 100.2±26.6 |  |
| **CI(L/min/m^2^)** | APRV | 3.23±0.88 | 3.94±1.69 | 4.71±1.07* | 4.08±0.64 | 4.96±1.55 | 5.32±0.79 | 4.85±0.81 | 4.11±0.78 |  |
|  | LTV | 2.89±0.58 | 3.67±1.24 | 2.82±0.77 | 3.34±1.06 | 3.48±1.34 | 3.68±1.43 | 3.32±1.24 | 3.56±1.46 |  |
| **HR(bpm)** | APRV | 61.0±9.8 | 78.8±31.6 | 101.5±8.3 | 94.0±21.4 | 109.8±26.6 | 102.0±15.6 | 87.5±15.8 | 87.8±17.1 |  |
|  | LTV | 62.2±10.8 | 75.0±21.2 | 72.4±11.9 | 87.2±28.8 | 100.2±39.3 | 86.6±17.2 | 89.2±32.7 | 94.2±47.3 |  |
| **Urine volume(ml)** | APRV | 400.0±264.6 | 990.0±429.3 | 1590.0±693.4 | 1999.0±741.3 | 2623.3±667.8 | 3198.3±674.6 | 3750.7±576.2 | 4209.0±599.3 |  |
|  | LTV | 347.5±238.2 | 620.0±358.6 | 1543.8±960.1 | 2119.8±882.3 | 2822.8±1057.5 | 3640.0±921.1 | 4351.5±911.2 | 4980.3.8±1373.5 |  |
| **Fluid resuscitation(ml)** | APRV | 833.3±208.2 | 2033.3±378.6 | 3316.7±76.4 | 4300.0±661.4 | 5353.3±408.1 | 6143.3±350.2 | 7010.0±385.1 | 7493.3±354.4 |  |
|  | LTV | 1200.0±282.8 | 2100.0±141.4 | 2615.0±374.8 | 4095.0±784.9 | 4735.0±586.9 | 5675.0±530.3 | 6765.0±120.2 | 7705.0±77.8 |  |
| **Norepi Dose (µg/kg/min)** | APRV | 0.00±0.00 | 0.00±0.00 | 0.41±0.39 | 0.62±0.72 | 0.73±0.74 | 1.00±0.49 | 1.38±0.79 | 1.32±0.16 |  |
|  | LTV | 0.00±0.00 | 0.00±0.00 | 0.00±0.00 | 0.57±0.36 | 1.00±0.69 | 0.88±0.79 | 0.94±1.03 | 1.67±2.28 |  |

*#* p<0.05 between groups with repeated measures analysis of variance

***p<0.05 between groups with t- test.

APRV, airway pressure release ventilation; LTV, low tidal volume; MAP, mean arterial pressure; CI, cardiac index; HR, heart rate
